# Allele frequencies of the human platelet antigen-1 in the Egyptian population

**DOI:** 10.1186/1756-0500-2-90

**Published:** 2009-05-20

**Authors:** Abdel Halim Salem, Kyudong Han, Mark A Batzer

**Affiliations:** 1 Department of Anatomy, College of Medicine and Medical Sciences, Arabian Gulf University, Manama, Kingdom of Bahrain; 2 Department of Anatomy, Faculty of Medicine, Suez Canal University, Ismailia, Egypt; 3 Department of Biological Sciences, Louisiana State University, Baton Rouge, LA 70803, USA

## Abstract

**Background:**

The human platelet alloantigen system HPA-1 in the Egyptian population was examined by polymerase chain reaction using sequence-specific primers (PCR-SSP). The objectives of this study were to evaluate the allele frequency of HPA-1a and -1b in healthy Egyptian individuals and compare these with the international literature. Human platelet antigen (HPA) systems are associated with alloimmunization and organ transplantation rejection as well as the development of cardiovascular disease. Of the various HPA systems, HPA-1 specifically has been considered to be the most important antigenic system implicated in the Caucasian population. No study has yet examined this system in the Egyptian populations, however. We therefore investigated the allele frequency of the HPA-1 system in the Egyptian population.

**Findings:**

To determine the allele frequency of the HPA-1a and -1b, we tested genomic DNAs from 206 healthy, unrelated Egyptian individuals using PCR-SSP. Our results showed that the 1a/1a genotype was the most predominant (59.22%) followed by 1a/1b (34.95%) and 1b/1b (5.83%) with allele frequencies for 1a and 1b of 0.77 and 0.23, respectively, in the population.

**Conclusion:**

As compared with other geographic groups, a relatively high allele frequency of the HPA-1b in the Egyptian population may indicate a higher risk of alloimmunization. This study is the first to investigate the allele frequency of the HPA-1 system in the Egyptian population and serves as an outline for future clinical research associated with platelet disorders in this group.

## Background

The human platelet antigens (HPA) systems derive from the single base pair substitution in the encoding genes of platelet membrane glycoproteins (GP). The GP variants resulting from amino acid substitutions are involved in the rate of alloimmunization to platelet-specific antigens. Subsequently, the alloimmunization can induce neonatal alloimmune thrombocytopenia (NAIT) [1], post-transfusion purpura (PTP), or platelet transfusion refractoriness (PTR) [2]. Therefore, accurate donor compatibility for platelet transfusions is extremely important. HPA systems are not only associated with organ transplantation rejection [3] and cardiovascular disease [4], but are also frequently assessed in general population studies. The molecular basis of the biallelic polymorphisms of all HPA systems (i.e. HPA-1, 2, -3, -4, -5, -15) is linked to platelet GP variants. The major GPs (GPIIb, GPIIIa, GPIb, and GPIa) generated by single amino acid substitutions are associated with various HPAs [5]. The presence of leucine or proline at position 33 of the GPIIIa results in two HPA-1a or HPA-1b antigens, respectively [6]. Therefore, molecular DNA-based analysis has been preferred for the HPA genotyping.

Recent studies of population genetics have reported that there is a heterogeneous diversity of HPA genotypes in different geographic groups. However, these studies have largely been performed in Asian, European, and North American populations [7-9]. Among Arabian populations, Egyptians are among the most centrally located to Africa, Europe, and Asia. This fact resulted in Egypt's varied cultural history and its population is a diverse genetic amalgam. To date, the HPA-1 polymorphisms have not been assessed in the Egyptian population. The aim of this study was to investigate the allele frequencies of HPA-1 and estimate the frequency of the 1a and 1b alleles among the healthy Egyptian population.

## Methods

### Samples and DNA extraction

Blood samples were collected from 206 unrelated, healthy Egyptians from Ismailia under institutionally approved internal review board protocols with informed consent. DNA was prepared from blood leukocytes by standard methods [10]. The genotypes of HPA-1 system were determined using the polymerase chain reaction sequence-specific primers (PCR-SSP) method designed by Skogen et al. and the SSPs were used to discriminate between the alleles encoding the six major HPAs in a series of patients and normal blood donors [11]. The thermocycler program consists of an initial step of 94°C for 5 min, followed by 32 cycles of 94°C for 30 s, 65°C for 60 s, 72°C for 60 s, and a final extension step of 72°C for 10 min. The PCR products (15 ul) were subjected to gel electrophoresis on standard 1.5% agarose gel containing 0.5 ug per ml of ethidium bromide. The typing results were examined under UV light transillumination. For each sample, it was possible to determine the absence or presence of the two alleles, 1a and 1b. Individuals are, therefore, genotypically classified as 1a/1a, 1a/1b or 1b/1b.

### Statistical analysis

Statistical analysis was performed using SPSS version 15 statistical package for windows. Allele and genotype frequencies were calculated by direct counting; Hardy-Weinberg equilibrium was assessed by an exact test provided by the Arlequin program [12].

## Results and discussion

The genotype and allele frequencies of HPA-1 system in the Egyptian population are shown in Table 1. The study population was found to be in Hardy-Weinberg equilibrium. The 1a/1a genotype is the most predominant (59.22%) followed by 1a/1b (34.95%) and 1b/1b (5.83%) (Table 1). In these 206 unrelated Egyptian individuals, the allele frequencies of 1a and 1b were calculated to be 0.77 and 0.23, respectively. Table 2 describes the allele frequencies at the HPA-1 with those previously reported in other ethnic populations.

**Table 1:** Distribution of the various HPA-1 genotypes and gene frequencies among the Egyptian population.

			**HPA-1 Allele Frequency**		**Heterozygosity Frequency**		**Total Chi^2 ^goodness of fit test**
				
**N**	**HPA-1 Genotype**	**Number Observed (Percentage)**	***1a***	***1b***	**Observed**	**Expected**	
206	*1a/1a*	122 (59.22)	0.767	0.233	0.350	0.357	0.101 (p = 0.7505)
	*1a/1b*	72 (34.95)					
	*1b/1b*	12 (5.83)					

**Table 2:** Allele frequencies of HPA-1a and -1b in different populations.

**Population [reference]**	**Allele Frequency (%)**		**N**
		
	**1a**	**1b**	
Berber Moroccan [22]	0.748	0.252	110
Tunisian [23]	0.750	0.250	90
Bahraini [21]	0.760	0.240	194
Egyptian [this study]	0.767	0.233	206
Lebanese [24]	0.810	0.190	205
Danish [28]	0.831	0.169	557
French [26]	0.848	0.152	800
Spanish [27]	0.851	0.149	500
Austrian [7]	0.852	0.148	911
Polish [25]	0.874	0.126	135
Caucasian-American [8]	0.890	0.110	100
African-American [8]	0.920	0.080	100
Korean [9]	0.988	0.012	200
Japanese [14]	0.998	0.002	331
Amazon Indian [29]	1.000	0.000	95

The allele frequencies of several of HPAs vary among different ethnic groups and their prevalence in a given population is a major determinant for the prevalence of HPA alloimmunization and its clinical associated entities: NAIT, PTP, PTR, post-transfusion passive alloimmune thrombocytopenia, and transplantation-associated alloimmune thrombocytopenia [13]. In one study, HPA-1 antibodies were found to be present in 80% of patients with NAIT, and the HPA-1a allele was concluded to be a factor contributing to the disease [1]. Although alloantibodies against the HPA-1 are frequently implicated in alloimmunization, the detection of HPA-1 is not recommended in African-American and Asian geographic groups because of the low allele frequency of the HPA-1b allele in these populations [8]. As shown in Table 2, the allele frequency of HPA-1b for the African-American population is in fact lower than that for the Caucasian-American population (0.080 vs. 0.110). Furthermore, antibodies against HPA-1 antigens were extremely rare in Japanese patients with NAIT or refractoriness to platelet transfusion, with an allele frequency of 0.002 for anti-HPA-1b [14]. Halle et al. recently studied four different sub-Saharan African populations (Beninese, Cameroonians, Congolese, and Pygmies) and the allele HPA-1b was reported to be somewhat low by contrast to other HPA systems (HPA-2, -3, -4, -5, -15) [15]. By contrast, the allele frequency of HPA-1b in Aka Pygmy populations was 0.000, a result that might explain the low risk of alloimmunization anti-HPA-1a compared with Caucasian populations [15].

An increasing number of population genetic studies have been conducted to assess the effect of the HPA-1 system on the risk of developing cardiovascular disease or myocardial infarction (MI). However, the data obtained to date contained contradictions, and the correlation is still under debate. Weiss et al. reported that HPA-1b increases the risk of MI or unstable angina in Caucasian populations [16]. The ratio of developing MI or unstable angina between patients with the HPA-1a/1a genotype and those with either the HPA-1a/1b or the 1b/1b genotype was found to be 6:2 [16]. Other studies highlighted the effect of HPA-1 in coronary artery disease and showed a positive correlation in the same age group [17], as well as in the Caucasian population [18]. By contrast, studies of the Japanese [19] and Korean [20] populations failed to demonstrate a significant correlation between HPA-1 and the risk of coronary artery disease or MI. Even in Caucasian populations, many studies did not detect any relationship [18].

As shown in Table 2, the Egyptians show an HPA-1 allele frequency that is similar to previously studied Arab populations (Tunisian, Moroccan, and Bahraini) except for the Lebanese [21-24]. The Egyptians have a slightly higher frequency of HPA-1b when compared to the Caucasian population (European and White American) [8,25-28]. The Berbers (Morocco) have been shown to have the highest frequency for the HPA-1b allele [22]. This is in contrast to the Asian populations and, in particular, the Amazon Indian population, which was shown to have total absence of the HPA-1b allele [29]. The relatively high allele frequency of the HPA-1b in the Egyptian population suggests that this ethnic group has a higher risk of alloimmunization. This is the first to study the allele frequency of the HPA-1 system in this population and, to date, there are no data available on the effect of HPA-1 on the risk of developing alloimmunization, cardiovascular disease, or MI among Egyptian patients. Although the prevalence of NAIT, PTP, and PTR has not been established in the Egyptian population yet, the detection of HPA alloantibodies before any transfusion or pregnancy should be recommended to prevent any clinical condition, especially when there is a positive family history of cardiovascular disease. This is because platelet hyperactivity caused by a conformational change in the GP receptors on the platelet surface may confer an increased risk for cardiovascular disease. The authors believe that this study will serve as a baseline for future clinical research associated with platelet disorders or cardiovascular diseases associated with the Egyptian population.

## Competing interests

The authors declare that they have no competing interests.

## Authors' contributions

AHS designed the research project. AHS performed the experiments and analyzed data. MAB contributed reagents/materials/analytic tools. KH performed part of the experimental procedures and helped to draft the manuscript. AHS and MAB wrote the manuscript. All authors read and approved the final manuscript.
